# Antioxidant Capacity, Nitrite and Nitrate Content in Beetroot-Based Dietary Supplements

**DOI:** 10.3390/foods12051017

**Published:** 2023-02-27

**Authors:** Joanna Brzezińska-Rojek, Svitlana Sagatovych, Paulina Malinowska, Kamila Gadaj, Magdalena Prokopowicz, Małgorzata Grembecka

**Affiliations:** 1 Department of Bromatology, Faculty of Pharmacy, Medical University of Gdansk, Al. Gen. J. Hallera 107, 80-416 Gdansk, Poland; 2 Student Scientific Circle at the Department of Bromatology, Faculty of Pharmacy, Medical University of Gdansk, Al. Gen. J. Hallera 107, 80-416 Gdansk, Poland; 3 Department of Physical Chemistry, Faculty of Pharmacy, Medical University of Gdansk, Al. Gen. J. Hallera 107, 80-416 Gdansk, Poland

**Keywords:** antioxidant capacity, beetroot, nitrate, nitrite, dietary supplement, CUPRAC, DPPH, Folin–Ciocalteu

## Abstract

Due to the high content of bioactive substances, beetroot and its preserves might be a valuable constituent of a diet. Research into the antioxidant capacity and content of nitrate (III) and (V) in beetroot-based dietary supplements (DSs) worldwide is limited. The Folin–Ciocalteu method, CUPRAC, DPPH, and Griess methods were used to determine total antioxidant capacity, total phenolic content, nitrites, and nitrates content in fifty DSs and twenty beetroot samples. Moreover, the safety of products was evaluated because of the concentration of nitrites, nitrates, and the correctness of labelling. The research showed that a serving of fresh beetroot provides significantly more antioxidants, nitrites, and nitrates than most daily portions of DSs. Product P9 provided the highest dose of nitrates (169 mg/daily dose). However, in most cases, the consumption of DSs would be associated with a low health value. The acceptable daily intake was not exceeded in the cases of nitrites (0.0015–0.55%) and nitrates (0.056–48%), assuming that the supplementation followed the manufacturer’s recommendation. According to European and Polish regulations, 64% of the products tested did not meet all the requirements for labelling food packaging. The findings point to the need for tighter regulation of DSs, as their consumption might be dangerous.

## 1. Introduction

Beetroot (*Beta vulgaris* L.) is a rich source of nutrients and bioactive substances such as fibre, carbohydrates, and phenolic compounds. In addition, this vegetable contains macro- and microelements such as potassium, iron, calcium, copper, sodium, and zinc, as well as vitamins B_1_, B_2_, B_3_, B_6_, biotin, and B_12_. Red beetroot owes its characteristic intense colour to betalain pigments-betacyanins: betanin (the dominant pigment), isobetanin, betanidin, isobetanidin, vulgaxanthin I and II, and indixanthin [1,2]. Beetroot peel has the highest betanin content. A correlation between antioxidant activity and the content of betacyanins has been found [3]. Betacyanins, along with phenolic acids, flavonoids, and ascorbic acid, are responsible for the antioxidant properties of beetroot. Furthermore, this vegetable is rich in nitrites and nitrates [4]. The oral bioavailability of nitrates from plants is 100% [5].

Beetroots might lead to several health-promoting effects, such as a stimulating effect on the circulatory and immune systems; improving the functioning of the endothelium; regulating the level of blood pressure; protecting the liver, the intestines, and the kidneys against toxic compounds; protecting against radiation consequences; and strengthening the gastric mucosa [4,6,7,8]. Due to these effects, the consumption of beetroot products may be beneficial in the cases of diabetes [9,10,11], post-menopausal women [11], diseases of the cardiovascular system [12,13], and athletes’ support [14,15,16]. Moreover, beetroot products with an appropriate concentration of inorganic nitrites can be an effective ergogenic agent, acting faster than a product containing only nitrate salts [17].

An excessive supply of nitrates may pose a health risk; therefore, in the interest of the health of consumers, a maximum acceptable daily intake (ADI) has been established that does not harm health when consumed throughout life. For nitrates, it is 0–5 mg/kg b.w. NO_3_¯ ions (corresponding to 0–3.7 mg NaNO_3_), while for nitrites, it is 0–0.2 mg/kg b.w. NO_2_¯ ions (corresponding to 0–0.07 mg of NaNO_2_) [18,19]. The main source of nitrates in the diet is vegetables. It is estimated that they provide 80–85% of the nitrates consumed. The supply of these compounds in drinking water, meat, or processed foods is much less important [5]. The total amount of nitrates consumed from all sources should be monitored, as there is a risk of exceeding the ADI, especially in children who have a lower body weight. Poisoning with nitrites may lead to methemoglobinemia or the development of neoplasms due to the formation of N-nitrosamines, which are carcinogenic. N-nitrosamines can be formed in the acidic environment of the stomach from nitrites in their reaction with secondary and tertiary amines [5].

Beetroot is consumed in various forms, such as fresh vegetables, juice, pickles, chips, and gel [20]. Dietary supplements containing *Beta vulgaris* L., manufactured in the form of tablets, lozenges, capsules, juices, powder, and many others, are also popular. However, producers often do not standardise products, which casts doubt on their effectiveness. Consequently, several potential risks for consumers appear, such as exposure to an excessive supply of nitrates or nitrites and loading the body with a product without health-promoting properties due to the lack of data on effectiveness compared to a fresh vegetable or the content of bioactive substances. A potential risk is also associated with the mislabelling of finished products.

Total antioxidant capacity (TAC) describes the antioxidant properties of a complex material (such as beetroot and beetroot preserves) consisting of numerous compounds. It is not just the sum of the antioxidant capacities of individual bioactive compounds. The TAC is the result of the synergistic effects of the different bioactive substances, trace elements, metals, vitamins, and other food constituents [21,22]. It was decided to determine the TAC instead of the concentrations of individual antioxidant substances because both the DSs and the vegetables are complex matrices, and their biological effect will be the result of the interaction of various components.

The research aimed to assess the quality and safety of beetroot-based DSs in comparison with beetroot samples. The TAC, total phenolic content (TPC), nitrites, and nitrate contents of fifty beetroot-based DSs (in the form of tablets, capsules, and powders) and twenty samples of beetroots available on the Polish market were determined for this purpose. Vegetables were divided into three subgroups: peeled, unpeeled, and skins. Reference was made to the average values for conventional and organic beetroots to compare DSs with vegetables. Manufacturers usually do not provide information on how the beetroot has been processed before manufacturing beetroot-based DSs. On several products, there was information that whole beetroot was used, which is why we also included vegetables with skins in our analysis. DSs are concentrated forms, so they can potentially provide a significant amount of bioactive substances, especially antioxidants and nitrogen compounds. As a result, the DS results were compared to vegetables to determine which are better sources of antioxidants, nitrites, and nitrates. The health risk was assessed because of the realisation of *ADI* for nitrites and nitrates. Furthermore, the correctness of the labelling of finished products was assessed based on Polish and European regulations because misinformation might also be dangerous for consumers. Statistical analyses were applied to verify the potential correlation between different methods of antioxidant capacity assessment and between TAC and nitrate and nitrite content. Moreover, it was assessed whether the content of antioxidants, nitrites, and nitrates differed statistically significantly in individual subgroups of beetroots. Despite the growing popularity of beetroot supplements, there is a lack of research on the quality and safety of their use.

## 2. Materials and Methods

### 2.1. Materials

#### 2.1.1. Sample Preparation 

**Figure 1:**
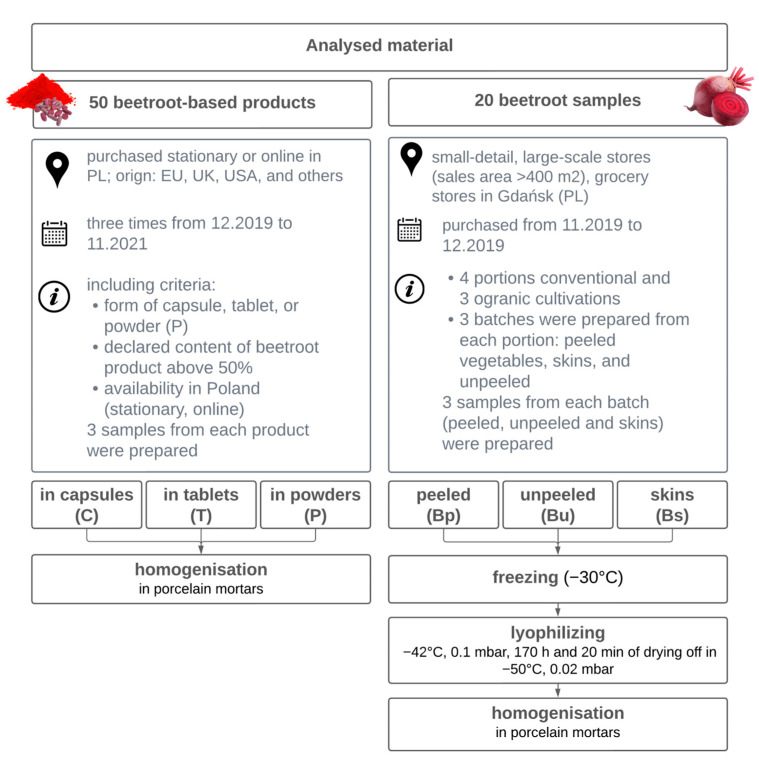
Scheme of collecting and preparation of samples.

The process of collection and sample preparation has been shown in Figure 1. Tables S1 and S2 provided in-depth details about beetroots and dietary supplements (DSs), respectively. Beetroot samples were lyophilized in an Alpha 1–4 LD plus freeze dryer (Christ, Osterode am Harz, Germany). Every DS was signed according to the alphanumeric code, including the formulation and the sequence number. Moreover, the letters (A, B, and C) were used to mark the same DSs with other serial numbers. To be more specific, ten of the examined products did not meet the requirements for labelling the category of dietary supplements, but they were tentatively included in the group of DSs in the following section of the work. At the purchase stage, they were described by sellers as “dietary supplements”. Only the verification of the labelling showed that they do not legally belong to this group, they are just traditional food products. They were marked in orange in Table S2. The exclusion criteria for beetroot-based DSs were: other forms of products (such as juice, shot, gel and bar); beetroot was not a main ingredient but an auxiliary substance; and unavailability for Polish consumers in the mentioned time period.

Only ceramic tools were used for sample preparation. In total, seventy samples of DSs and vegetables were analysed in triplicate.

#### 2.1.2. Reagents and Standards

Reagents for the Folin–Ciocalteu assay were as follows: anhydrous sodium carbonate (purity > 99.5%, Chempur^®^, Piekary Slaskie, Poland), Folin–Ciocalteu reagent (analytical grade, Chempur^®^, Piekary Slaskie, Poland), gallic acid (Sigma-Aldrich^®^, Darmstadt, Germany). Reagents for the CUPRAC assay were as follows: ethanol 96% (LiChrosolv^®^, Darmstadt, Germany), 6-Hydroxy-2,5,7,8-tetramethylchromane-2-carboxylic acid (Sigma-Aldrich^®^, Buchs, Switzerland), copper (II) chloride (Sigma-Aldrich^®^, St. Louis, MO, USA), ammonium acetate (Sigma-Aldrich^®^, Darmstadt, Germany), neocuproine (Sigma-Aldrich^®^, Darmstadt, Germany). Reagents for the DPPH assay were as follows: methanol (LiChrosolv^®^, Darmstadt, Germany), 2,2-diphenyl-1-picrylhydrazyl (Sigma-Aldrich^®^, Darmstadt, Germany).

Reagents for the nitrites and nitrates determination were as follows: hydrochloric acid 35–38% (Chempur^®^, Piekary Slaskie, Poland), acetic acid min. 99.5% (Chempur^®^, Piekary Slaskie, Poland), sodium nitrite (Chempur^®^, Piekary Slaskie, Poland), sodium tetraborate (POCH, Gliwice, Poland), sulfanilamide (Sigma-Aldrich^®^, Darmstadt, Germany), N-naphthylethylenediamine dihydrochloride (Sigma-Aldrich^®^, St. Louis, MO, USA), zinc acetate dihydrate (POCH, Gliwice, Poland), Carrez solution I—Potassium hexacyanoferrate (II) 0.25 mol/L (aqueous solution) (VWR, Leuven, Belgium), ammonium buffer pH 9.6 (obtained from ammonia (POCH, Gliwice, Poland) and hydrochloric acid 37% (VWR, Leuven, Belgium), cadmium sulphate (VI) (Chempur^®^, Piekary Slaskie, Poland), zinc sticks ≥99.99% (Merck Millipore, Darmstadt, Germany).

Ultrapure water (18.2 MΩ·cm, Millipore Simplicity System, Billerica, MA, USA) was used for all aqueous solutions.

### 2.2. The Total Antioxidant Capacity (TAC) and Total Phenolic Content (TPC)

The assessment of the TAC was carried out using the CUPRAC and DPPH assays. Moreover, the Folin–Ciocalteu (FC) method was applied to determine TPC.

#### 2.2.1. Extract Preparation for TAC and TPC Determination

**Figure 2:**
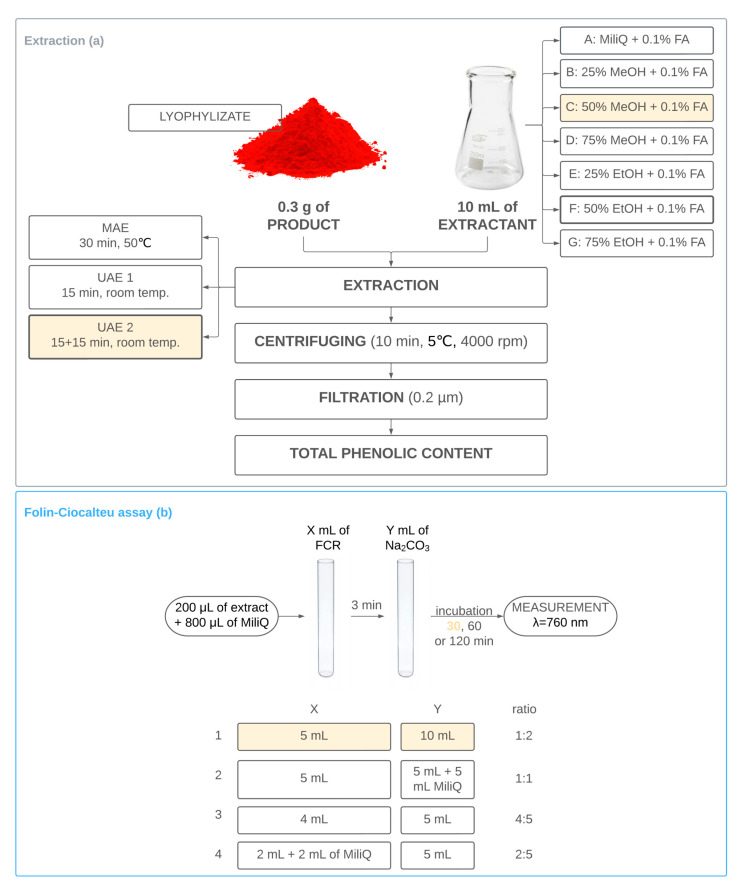
Diagram showing the optimisation of the samples’ extraction (**a**) and the Folin–Ciocalteu method (**b**). The selected conditions are marked in yellow in the figure.

Based on the conditions described by Capanoglu et al. [23] and other literature reports on the extraction of beetroot products [24,25,26,27,28], optimisation of the extraction was performed using seven combinations of solvents in three variants of extraction (Figure 2). Each variant was examined in triplicate with a threefold measurement. Variant C was chosen as the most optimal (based on results of ANOVA test): two-stage ultrasound-assisted extraction with 50% MeOH + 0.1% FA (marked in yellow in Figure 2; *p* < 0.05). Table S3 summarises the obtained results. The extraction was carried out on the selected lyophilizate; therefore, the results are expressed in mg GAE/g of lyophilizate.

**Figure 3:**
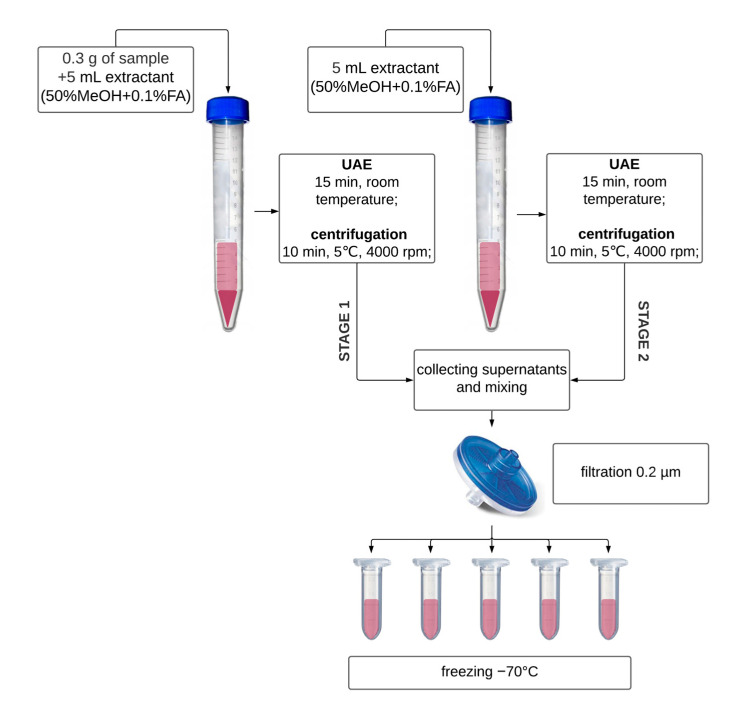
The procedure of extraction for TAC and TPC determination.

Figure 3 depicts the extraction procedure. Each sample was examined in triplicate with a threefold measurement. Lyophilizates and DSs were homogenized in a mortar straight before analyses. The samples were kept in the freezer all of the time and were thawed at room temperature before starting the individual analyses.

#### 2.2.2. TPC Determination

The total phenolic content (TPC) in the extracted samples was determined using the Folin-Ciocalteau reagent (FCR), according to the optimised and validated method developed by the authors based on literature research [20,29,30,31,32,33,34]. The mutual ratio of the reagents used (FCR and Na_2_CO_3_) and the incubation time before the measurement were optimised according to the scheme shown in Figure 2. Each variant was examined in triplicate with a threefold measurement. The obtained results are summarised in Tables S4 and S5. Model 1 was found to be the most efficient (ANOVA; *p* < 0.05), thus, 5 mL of FCR was mixed with 10 mL of Na_2_CO_3_, and 30 min of incubation was applied. It was assumed that the composition of supplements in the form of tablets may differ the most from pure lyophilizate due to the presence of auxiliary substances enabling the tabletting process; thus, the time of incubation was also optimised for the product in a tablet.

Sample extracts (x mL) and Mili-Q water ((1.0 − x) mL) were placed in a centrifuge tube to have a volume of 1 mL. Next, 5 mL of FCR was added, the sample was mixed, and it was left for 3 min. Then, 10 mL of saturated sodium carbonate solution (150 g/L) was added. The test tubes were carefully blended after the addition of each reagent using a vortex (Lab dancer, VWR^®^, Gdansk, Poland). The absorbance was measured threefold at 760 nm (Genesys 10S, Thermo Fisher Scientific, Waltham, MA, USA) after incubation (30 min at room temperature without light). Results were expressed in gallic acid equivalents (mg GAE/g of product and mg GAE/daily dose of product for DSs or mg GAE/g dry weight (d.w.) and mg GAE/100 g fresh weight (f.w.) of beetroot).

Analogously, a calibration curve was prepared to range from 0.1 to 10 µg/mL. The calibration curve was made in three independent replications with threefold measurements.

#### 2.2.3. CUPRAC

The CUPRAC assay was carried out as described by Apak et al. [35]. Analogously, a calibration curve was prepared in the range of 0.0005 to 0.07 uM/mL of Trolox. Three independent replications with threefold measurements were used to create the calibration curve. Results were expressed in Trolox acid equivalents (TE) (µmol TE/g of product and µmol TE/daily dose of the product, or µmol TE/g d.w. and µmol TE/100 g f.w. of beetroot). The incubation time of the samples was previously optimised. Results after 30 min and 60 min of incubation did not differ significantly, so the first one was applied.

#### 2.2.4. DPPH

The DPPH assay was carried out as described by Ravichandran et al. [36]. The absorbance of the sample was measured threefold at 515 nm (Genesys 10S, Thermo Fisher Scientific, Waltham, MA, USA) after incubation (30 min at room temperature). Results were expressed as a percentage of the antioxidant activity, which was calculated as follows:

(1) $$\mathrm{Activity}\;\left(\%\right)=\frac{\mathrm{Ac}\;-\;\mathrm{As}}{\mathrm{Ac}}\times 100\%$$

Ac—absorbance of control;

As—absorbance of a sample.

### 2.3. The Nitrite and Nitrate Determination

Quantification of nitrites and nitrates in beetroot (*Beta vulgaris* L.) and beetroot products was carried out by spectrophotometry using Griess reagents I, II and III according to ISO 6635-1984 (E) [37].

#### 2.3.1. Extraction for Nitrites and Nitrates Determination

A total of 1.0 to 10 g of the test sample were weighed, according to the expected nitrite content. Then, 3.0 g of activated carbon, 5 mL of disodium tetraborate solution, and 100 mL of hot, purified water were added to each sample. The flasks were shaken for 15 min at 80 °C. Next, 2 mL of potassium hexacyanoferrate (II) and 2 mL of zinc acetate solution were added to the samples. The solutions, after cooling to room temperature, were transferred to 200 mL volumetric flasks, made up to the mark, and shaken. Finally, solutions were filtered into conical flasks through paper filters.

#### 2.3.2. Nitrites Determination

At least 10 mL of solution was transferred to the 50 mL volumetric flask and diluted into 30 mL with purified water. Then, 5 mL of solution I (sulfanilamide dissolved in water with hydrochloric acid) and 3 mL of solution III (hydrochloric acid) were added. The content of the flask was thoroughly mixed and left for 1 min at ambient temperature, protected from light. Next, 1 mL of solution II (0.1% solution of N-(1-naphthyl)ethylenediamine dihydrochloride) was added, mixed carefully, and left for 3 min at ambient temperature, protected from light. After making up to the mark with water, the solution was mixed. The absorbance at a wavelength of 538 nm was measured within 15 min using the spectrometer. Results were expressed as µg/g of NO_2_¯ and µg/daily dose of NO_2_¯ or µg/g d.w. of NO_2_¯and µg/100 g f.w. of NO_2_¯ of beetroot, which is calculated as follows:

(2) $$\left({\mathrm{NO}}_{2}^{-}\right)=m_{1}\times \frac{200}{V_{1}\times m_{0}}$$

$m_{0}$—the mass, in grams, of the test portion;

$m_{1}$—the mass, in micrograms, of nitrite ion (NO_2_¯) contained in the aliquot portion ($V_{1}$) of filtrate taken, read from the calibration graph;

$V_{1}$—the volume, in millilitres, of the aliquot portion of filtrate taken.

Analogously, a calibration curve was prepared to range from 0.0 to 0.06 µg/mL of nitrites. The calibration curve was made in three independent replications with threefold measurements.

#### 2.3.3. Nitrates Determination

About 2 g of the cadmium and 5 mL of the buffer solution, and an aliquot portion of the filtrate (10 mL or less) were placed in a 25 mL conical flask. The flask was agitated for 5 min. Next, the solution was filtered into a 50 mL one-mark volumetric flask and made up to the mark. The determination proceeded analogously to total nitrites (Section 2.3.2) using 10 mL of the test solution. Results of nitrate determination were expressed as µg/g of NO_3_¯ and µg/daily dose of NO_3_¯ or µg/g d.w. of NO_3_¯and µg/100 g f.w. of NO_3_¯ of beetroot, which was calculated as follows:

(3) $$\left({\mathrm{NO}}_{3}^{-}\right)=1.348\left(\frac{m_{2}\times 10\;000}{V_{3}\times V_{2}\times m_{0}}-\frac{m_{1}\times 200}{V_{1}\times m_{0}}\right)$$

$m_{2}$—the total mass of nitrite, in micrograms of nitrite ion (NO_2_¯), contained in the volume ($V_{2}$) of test solution taken, read from the calibration graph;

$V_{2}$—the volume, in millilitres, of the test solution taken for the spectrometric measurement;

$V_{3}$—the volume, in millilitres, of the aliquot portion of the filtrate taken for the preparation of the test solution;

$m_{0},\;m_{1},\;V_{1}$—have the same meanings as in Equation (2).

The ratio between the relative molecular masses of the nitrate ion (NO_3_¯) and nitrite ion (NO_2_¯) is 1.348.

### 2.4. Validation

The following validation parameters were determined for all methods: linearity range, precision, accuracy, the limit of determination (LOD), and the limit of quantification (LOQ). The LOD and LOQ were computed as described by Huber [38]:

(4) $$LOD=\frac{3.3SD_{a}}{b}$$

*SDa*—standard deviation of the intercept for the calibration curve;

*b*—slope for the calibration curve.

(5) LOD=3×LOD

Table 1 shows the results of the validation. Due to the lack of reference material corresponding to the analysed material, accuracy was determined using the method of standard addition (GA in the FC assay and Trolox in the CUPRAC assay) to the chosen DS and lyophilizate and was expressed as recovery. DPPH assay was validated based on gallic acid standard solutions. The average recovery for the selected methods was in the range of 80–120% which was an acceptable level for such an analysis. The precision was computed as the coefficient of variation for all the results obtained in all the analysed samples during validation. The signal obtained for standards (S_expected_) and the signal calculated from the calibration equation (S_calculated_) were applied for the calculation of recovery for calibration curves (*Rcc*):

(6) $$Rcc=\frac{\left\lfloor S_{\mathrm{expected}}-{\;S}_{\mathrm{calculated}}\right\rfloor }{S_{\mathrm{expected}}}$$

### 2.5. Labelling Assessment

Thirty-four packages of DSs and eight traditional food products were assessed. Before evaluating packaging and labelling, each product’s registration in the register of products subject to notification of first market placement was checked [39]. As a result, eight products could not be included in the “dietary supplement” category due to the lack of appropriate labelling on the packaging and registration with the Chief Sanitary Inspectorate (GIS). Requirements on food and DS labelling are specified in Regulation (EU) No 1169/2011 [40] and the Act on Food and Nutrition Safety of 25 August 2006 [41]. As food, DSs have been assessed because of the requirements specified in the Regulation of the Minister of Health of 9 October 2007 [42]. The correctness of the labelling was assessed according to the following criteria [39,40,41,42]:

- Labelling in Polish;
- The name of the food;
- The list of ingredients;
- The net amount of food;
- The date of minimum durability or best-before date;
- The presence of the term “dietary supplement”;
- Indication of the recommended daily portion of the product;
- The presence of a warning regarding not exceeding the recommended daily portion;
- A statement that dietary supplements cannot be used as a substitute for a varied diet;
- A statement that they should be kept out of the reach of small children.

In addition, the manufacturer should provide information on the content of active ingredients per recommended daily portion and information on the content of vitamins and minerals in percentages concerning the reference daily intake (RDI). Particular attention was paid to the health claims on the packaging of the tested products, which were compared with the list of permitted health claims defined in Regulation No. 1924/2006 [43,44] and with the statements contained in the register of the European Food Safety Authority [45]. The difference in the number of analysed packages versus the total number of analysed DSs is due to the fact that some products were purchased in multiple repetitions (different lot numbers), resulting in the same package design.

### 2.6. Statistical Analyses 

The data were reported as the mean ± standard deviation of three independent samples, each measured three times. Statistical analyses such as the ANOVA Kruskal–Wallis test, the U Mann–Whitney test, or Spearman’s rank correlation coefficient preceded by an analysis of the normality (the Shapiro–Wilk test) of the distribution were used to compare the treatments. They were performed by the Statistica for Windows (version 13, Statsoft, Cracow, Poland) software package. Differences at *p* < 0.05 were deemed significant. The validation parameters for spectrophotometric assays, the overall mean, and the standard error values were calculated using the Microsoft Office Excel software (version 2007 12.0.6787.5000 SP3 MSO, Microsoft Corporation, Redmond, WA, USA).

## 3. Results

### 3.1. Total Phenolic Content and the TAC

The averaged values of TAC (CUPRAC, DPPH) and TPC (FC) in the analysed beetroot and DSs, divided into groups (tablets, capsules, powders), are shown in Table 2 and Table 3, respectively. For the FC, results are expressed in gallic acid equivalents (GAE), for CUPRAC in Trolox equivalents (TE), and for DPPH as a percentage reduction in DPPH. Tables S6 and S7 show the full characteristics of the beetroot and beetroot-based DSs studied due to their TAC, TPC, nitrate, and nitrite contents. Powders were characterised by significantly higher TPC (mg GAE/d. d.; Table S8) than tablets (U Mann–Whitney test, *p* = 9.9 × 10^−5^) and capsules (U Mann–Whitney test, *p* = 4.4 × 10^−5^). However, lyophilizates showed the highest TPC compared to any group of DSs. There was no statistically significant difference between tablets and capsules (U Mann–Whitney test, *p* = 0.99). In the case of TPC per gram of a product, a statistically significant difference was found only between tablets and lyophilizates (U Mann–Whitney test, *p* = 0.0049), which could be caused by the presence of excipients in tablets used in tabletting processes. In the FC assay, the product marked as T7 (41 mg GAE/d. d.) was characterised by the highest TPC among the tablets, C8A (42 mg GAE/d. d.) and C8B (41 mg GAE/d. d.) among the capsules, and product P9 (251 mg GAE/d. d.) among the powders.

Powders showed higher TAC than tablets (U Mann–Whitney test, *p* = 1.6 × 10^−4^) and capsules (U Mann–Whitney test, *p* = 2.3 × 10^−5^). Lyophilizates provided a higher TAC than DSs. Considering TAC expressed as TE/g, lyophilizates showed significantly more antioxidants than all DS formulations. The highest TAC among the tablets was found in the T7 product (350 μmol TE/d. d.), the C13 (363 μmol TE/d. d.) among capsules, and the P9 product (3520 μmol TE/d. d.) among the powders. It is worth noting that product P9 exhibited a higher TPC (251 mg GAE/d. d.) and TAC (3520 μmol TE/d. d.) than average beetroots (Table 2). Different trends between the FC and CUPRAC methods may result from the variability of the conditions under which the tests were conducted and the reaction mechanisms. In the CUPRAC method, for example, the antioxidant potential is tested at pH = 7, which is close to the pH of human blood, as opposed to the FC method, which tests at pH 8–9. These changes in pH can influence the development of various antioxidant capacities of products, especially considering the complex matrix of DSs. In addition, the reaction with the DPPH radical is specific to individual antioxidants; they can react at different rates. Despite the concentrated DS formula as dried material, the daily portion of fresh beetroot (100 g f.w.) was richer in antioxidants.

In the DPPH method, the highest TAC was shown by the product T7 (90%) among the tablets and the products C6 and C8B among the capsules—the activities of which were both 90%—while the activity of the product C8A was 74%. Among the powders, the product P11 (90%) showed the highest TAC. The content of antioxidants in the dietary supplements T2A, T2B, and T2C was greatly varied, despite the use of the standard material standardisation procedure. Moreover, all three supplements were sold as the same commercial product. Three other products were characterised by a high RSD (30% for T6; 41% for T10; and 33% for C2). In all cases, the analysis was repeated, but the analogous results were obtained, and the Q-Dixon test did not show a significant error. A high RSD could be caused by the heterogeneity of the supplement, as the method has been validated and the analysis conditions have not changed.

In a study conducted by Guldiken et al. [20], in which the content of antioxidants was measured using colorimetric methods in fresh beetroot, the following results were obtained: 255 mg GAE/100 g of fresh weight in the FC method and 15,538 μmol TE/100 g (3889 mg TE/100 g) in the CUPRAC method. The values obtained by the FC method are comparable to those obtained in this work for the majority of whole and peeled beetroot lyophilizates (Table S7). However, the values obtained by the CUPRAC method in this work are lower for most samples. Only the samples of skins 6Sk and 7Sk, which were from organic farming, can be considered comparable (9779 and 8932 μmol TE/100 g, respectively). This may be due to the differences in the profile of compounds and antioxidant capacity between different varieties and the freshness of the material analysed. In this study, the vegetables were processed and freeze-dried immediately after purchase. However, there is no way to trace the storage conditions of fresh material before purchase. There is a lack of reports in the literature regarding the TPC and TAC of DSs made from beetroot. Comparing the results obtained for supplements per gram in capsules (0.68 to 33 mg GAE/g), tablets (2.0 to 41 mg GAE/g) or powders (4–61 mg GAE/g) with the results obtained by Guldiken et al. [20] for dried beetroot 3.3 mg GAE/g (347 mg GAE/100 g), there can be observed a lower TPC in dried beetroot compared to our supplements calculated per g of d.w.

Moreover, Spearman’s rank correlation coefficient was performed to test for a potential correlation between the antioxidant potential results obtained by different methods. A fairly strong relationship (0.7–0.9) or a very strong relationship (>0.9) was observed between the results obtained by the FC, CUPRAC, and DPPH methods in all the groups analysed (beetroots, capsules, tablets, and powders) (Table S9). In the study by Apak et al. [46], the correlation between the CUPRAC and FC methods was comparable and amounted to r = 0.966. Another study by Güçlü et al. showed a high correlation (r = 0.93) between the FC and CUPRAC methods [47].

### 3.2. Nitrate and Nitrite Content

In general, significantly lower levels of nitrite ions (0.21–78 µg/d. d.) than nitrate ions (0.197–169 mg/d. d.) were found in DS samples. Due to the lack of a normal distribution (the Shapiro–Wilk test, *p* < 0.05), the U Mann–Whitney test was applied to check for statistically significant differences. It was found that supplements in tablets (*p* = 0.000295) and capsules (*p* = 0.014038) contained significantly fewer nitrite ions, as well as supplements in tablets (*p* = 0.262612) had statistically significantly fewer nitrate ions than lyophilized vegetables, considering their content in 1 g of product (dry weight for beetroot). The nitrite ion content of powders and beetroots did not differ significantly, nor did the nitrate ion content of beetroots, capsules, and powders. Moreover, individual parts of beetroot (peeled beetroot, skins) did not differ significantly in the content of nitrites (*p* = 0.133615) and nitrates (*p* = 0.830324) (the U Mann–Whitney test, *p* < 0.05). All results of the U Mann–Whitney test were shown in Table S10. An average portion of conventional beetroots provided more nitrites (49 µg/100 g f.w.) and nitrates (90 mg/100 g f.w., Table 3) than most of the other products analysed. Only products P9 (169 mg/d.d.), P10 (99 mg/d.d.), and P13 (131 mg/d.d.) contained more nitrates than beetroots. The highest content of nitrite ions was found in supplement number C4 (8.4 µg/g) in the case of capsules, T3A (2.48 µg/g) for tablets, and P13 (6.4 µg/g) for powders. The highest level of nitrate ions was found in T11 (3746 mg/kg), C16 (15,186 mg/kg), C7B (11,924 mg/kg), P13 (13,110 mg/kg), and P9 (10,224 mg/kg) DSs.

In all tested vegetable samples, a significantly lower content of nitrite ions (0.702 µg/g–15 µg/g) than nitrate ions (423 mg/kg-8801 mg/kg) was determined. The highest content of nitrite ions among vegetables was found in skin samples, except for group 7, where the highest level of these ions was determined in a sample of peeled beetroot. In the case of nitrate ions, the situation was the opposite: the skin samples were characterised by the lowest content of these ions, except for sample no. 1, where their level was the highest in the batch. Moreover, individual subgroups of beetroot (peeled, unpeeled, skins) differed in terms of TAC and TPC, regardless of the method used to assess the potential (ANOVA Kruskal–Wallis test: FC *p* = 0.0022, CUPRAC *p* = 0.0016, DPPH *p* = 0.006). The skins were the richest in antioxidants (Dunn’s test, *p* < 0.05). There were no statistically significant differences in the content of nitrites and nitrates between the individual subgroups (ANOVA, *p* > 0.05). For the comparison of vegetable supplements, reference was made to the average values for conventional and organic beetroots (Table 2). Manufacturers usually do not provide information on how the beetroot has been processed before preparing supplements from it. Several products contained information that whole beetroot was used, which is why we also included vegetables with skin in our analysis. The content of these compounds in beetroot depends primarily on the amount of nitrogen fertilization, agrotechnical treatments, and the plant growth phase [48]. Gościnna and Czapski [48] observed higher contents of nitrates in the middle parts of the root compared to its outer parts. Although they used a different division of the tuber (into 4 parts), it can be considered that the conclusions from our study and their research are consistent-beetroot skin is characterised by a lower content of nitrates.

Only four DS had a nitrate content declaration. Supplements C10 and C11 did not contain nitrates (<LOQ) despite their presence being declared by the manufacturer. Both products were produced by the same manufacturer but were available under different trade names and with different graphic designs. Product C1 contained a negligible amount of nitrates compared to the declaration (4.2%). Product P9 contained nitrates in amounts close to the declared one (85%). Simultaneously, it is the product that contains the most nitrates per daily portion of all the tested foods, as well as more than the average portion of fresh beetroot.

In the years 2003–2004, research on the content of nitrites and nitrates was carried out on certain vegetables purchased in random shops in Olsztyn [49]. Among these vegetables, beetroot was included, which was classified as a plant with a high content of nitrate—an average of 1408.17 mg/kg. A high level of nitrates (III) was determined in the analysed beetroots (on average 11.4 mg/kg), which differed from the average values for this vegetable. The content of nitrite and nitrate ions in the beetroot samples in this study was 0.120–2.935 mg/kg for nitrites and 102.30–1619.80 mg/kg for nitrates, respectively.

#### Health Risk Assessment

In terms of nitrite content (2.1% ADI for NO_2_¯), none of the products tested posed a risk (Table 4). Fresh beetroot (100 g) provided more NO_3_¯ (15–20.1% ADI for NO_3_¯, conventional and organic, respectively) than any of the analysed DSs in the form of tablets (3.2% ADI for NO_3_¯) or capsules (5.1% ADI for NO_3_¯). DSs in powders provided a similar amount of the substance as a serving of beetroot (based on the realisation of the ADI). Product P9, marketed by the manufacturer as having an “increased dose of nitrates”, had the highest nitrate dose (48% of the ADI for NO_3_¯) and the lowest nitrite levels (0.21% of the ADI for NO_2_¯). That is why the manufacturer advertised it as a product for athletes to be consumed before and after training “to increase the body’s efficiency, accelerate regeneration after training, and reduce accumulated lactic acid” (information on the packaging). In comparison, the recommended daily dose of products in capsules provided a maximum of 3.2% ADI for NO_3_¯ and 5.1% ADI for NO_3_¯ in tablets.

Keller et al. [50] analysed eighteen DSs in terms of the content of nitrites and nitrate and determined the percentage of ADI to evaluate the exposure to these compounds through the intake of the recommended portion. The ADI for nitrate amounted to 22% in the case of the Neo40 supplement, described by the manufacturer as “improving the functioning of the cardiovascular system”, and 97% in BeetElite—advertised as “improving exercise endurance and increasing oxygen supply in the body. The ADI for nitrites amounted to 450% and 225%, respectively. The rest of the DSs were characterised by values within the range of 0.01–47.26% for nitrates and 0.00–21.11% for nitrites [50]. The reason for such a high content of nitrites and nitrates in some supplements was most likely their composition—rich in nitrates dehydrated or concentrated forms of vegetables or concentrated vegetable juices. The supplements analysed for this work did not pose a risk to the consumer because of the ADI.

Studies have shown [50] that consumption of beetroot products is more beneficial than supplementation with nitrate salts because the flavonoids and vitamin C present in beetroot reduce the risk of nitrosamine formation. Haem iron may increase the risk of the formation of these compounds [50]. However, beetroot supplements, if fortified with iron, are in the non-haem form (usually iron gluconate or fumarate; see T2, T5, and T6). Because of ADI limits, all sources of nitrates in the diet should be considered when estimating daily nitrate and nitrite consumption and performing a safety assessment. Green leafy vegetables and root vegetables such as beetroot constitute rich sources of these substances [51,52]. Moreover, nitrates are added to meat and meat products to prevent *Clostridium botulinum*, *Listeria monocytogenes*, *Bacillus cereus*, *Clostridium perfringens*, and *Staphylococcus aureus* growth, improve their colour, and develop their characteristic flavour [19,53,54]. According to some studies, their high concentrations in water (>50 mg/L) can cause methemoglobinemia and gastrointestinal carcinogenesis [55]. It should be mentioned that hypotensive effects were observed after the nitrate dose corresponding with the upper limit of the WHO ADI. Ashor et al. [56] described the hypotensive effect after using beetroot juice rich in NO_3_¯ (70 mL containing 400 mg). Similarly, Mills et al. [57] discovered a hypotensive effect after 6 months of drinking beetroot juice rich in NO_3_¯ (70 mL containing 694 mg of NO_3_¯). Furthermore, Kapil et al. [58] reported that 4 weeks of supplementation with beetroot juice containing 450 mg of NO_3_¯ had beneficial therapeutic effects on endothelium and arterial stiffness. However, no therapeutic effects were observed with additional daily administration of 300 mg of NO_3_¯ [59,60]. Considering these values, the tested supplements are probably not able to exert a hypotensive effect even with long-term use, as the highest content of nitrates found in product P9 amounted to 48% of ADI.

### 3.3. The Correlation between the Antioxidant Potential and the Content of Nitrites and Nitrates in Beetroot and Beetroot–Based Products

A statistically significant negative correlation (Spearman’s rank correlation analysis) was found only in beetroot samples, both between the results of the antioxidant potential obtained by the FC and CUPRAC methods and the content of nitrates (Table S11). The group of beetroot samples was more homogeneous in terms of composition than DSs. Some of the DSs were enriched in various substances such as nitrates, iron compounds, and vitamin C, which could have disturbed the existence of a potential correlation. The Spearman’s rank correlation coefficient equals −0.54 for the FC method and −0.62 for CUPRAC, which means that dependence is moderate.

### 3.4. Labelling Assessment

An assessment of thirty-four packages of dietary supplements and eight traditional food products was carried out because of Polish and European food labelling regulations. It was found that 64% of packaging did not meet the legal requirements for food labelling, 12% were not reported to the Chief Sanitary Inspectorate, and 6% did not have the term “dietary supplement” on the packaging, despite having registration in the GIS in this category. Furthermore, 26% of products were not fully labelled in Polish, as a result of which the consumer is not able to get acquainted with the information presented on the packaging in detail and its content is not formulated understandably. It is worth emphasising that 21% of the tested products contained prohibited health or non-registered claims, which means that 15% of the products suggested that they had the properties of preventing or treating diseases. It is also worth noting that the product P9, which contained the highest amount of nitrates and was sold as a supplement, contained significant labelling deficiencies, including the lack of the wording “dietary supplement”. Moreover, part of the labelling was only in English, and the dosage was not precisely defined, which poses a risk of nitrate overdose.

The detailed results of the analysis are summarised in Table 5.

## 4. Conclusions

The importance of this study was to determine and compare TAC, TPC, nitrite, and nitrate content in beetroot-based DSs and beetroot. Moreover, the safety of consumption of DSs because of nitrites, nitrates, and the correctness of labelling were assessed.

The research revealed that TAC, TPC, nitrate, and nitrite concentrations expressed per unit of product weight (g or kg), DSs in capsules, and DSs in powders were comparable to the average beetroot. Tablets contained notably fewer of these substances, which might result from the presence of auxiliary substances used for tabletting. However, the average portion (100 g) of conventional or organic beetroot provided significantly more nitrates, nitrites, and substances with antioxidant properties than most of the DSs in capsules, tablets, and powders dosed according to the manufacturer’s recommendations. Only P9 (48% ADI for NO_3_¯), P10 (28% ADI for NO_3_¯), and P13 (37% ADI for NO_3_¯) delivered higher doses than beetroots. Most of the products did not have the declared content of nitrates. The antioxidant content in a serving of tablets or capsules was negligible, so their use has a low health value. In many of the samples studied, the nitrate content was not correlated to the antioxidant potential. A statistically significant negative correlation was found only in beetroot samples between the results of the FC and CUPRAC methods and the content of nitrates.

The labelling assessment has shown that 64% of packaging did not meet the legal requirements for food labelling. Some DSs contained illegal health claims that suggested healing properties or were misleading. This situation might result in reduced effectiveness or withdrawal from conventional therapies by consumers who would choose adulterated DSs. There were significant deficiencies in labelling, including a lack of full labelling in Polish, unclear dosage and others. Such deficiencies, combined with unknown nitrite and nitrate content, may result in consumers overdosing on these substances as a result of incorrect product intake. In addition, the unknown content of nitrates and nitrites may pose a threat to the consumer because the content of these compounds in vegetables varies depending on the place of origin and growing conditions. The conducted research indicated a strong need for more rigorous control when launching DSs, both in terms of composition and labelling. Although production and labelling guidelines are published, there is a lack of decisive action by national and European authorities related to the control and withdrawal of defective products.

## Figures and Tables

**Table 1:** The validation parameters of the applied methods.

	CUPRAC		FC		DPPH	Nitrates and Nitrites	
Standard substance	Trolox (TE)		gallic acid (GA)		gallic acid (GA)	sodium nitrite	
Calibration curve equation	y = 17.3x + 0.00234		y = 0.131x + 0.000874		y = −0.481x + 0.786	y = 1.0973x + 0.0037	
The determination coefficient (R^2^)	0.9993		0.9991		0.9991	0.9998	
Linearity range	0.0005–0.070 μmol/mL		0.10–10 μg/mL		0.13–1 μg/mL	0.0027–0.6 µg/mL	
LOD	0.000187 μmol/mL		0.074 μg/mL		0.040 μg/mL	0.009 μg/mL	
LOQ	0.000562 μmol/mL		0.22 μg/mL		0.12 μg/mL	0.0027 μg/mL	
	supplement	lyophilizate	supplement	lyophilizate	gallic acid	supplement	lyophilizate
Precision	1.7–3.1%	0.34–1.4%	1.1–3.9%	1.1–5.5%	2.0–4.6%	I stage: 1.26–1.97% II stage: 4.73–4.91%	I stage: 0.22–4.28% II stage: 2.11–4.95%
Recovery	95–115%	99–109%	96–106%	86–105%	91–113%	I stage: 86.17–93.47%II stage: 99.06–104.07%	I stage: 80.77–94.08%II stage: 93.99–104.72%

**Table 2:** Results of TAC, TPC, nitrite, and nitrate content in analysed conventional and organic beetroots.

	Beetroot												
Method	Unit	Conventional						Organic					
		n	Mean	SD	Min	Median	Max	n	Mean	SD	Min	Median	Max
FC	mg GAE/g d.w.	12	15	0.702	6.8	12	26	8	14	0.47	9.7	13	34
CUPRAC	µmol TE/g d.w.	12	171	11	93	150	365	8	196	7.1	123	179	413
Nitrites	µg/g d.w.	12	2.4	0.25	0.702	1.8	7.1	8	5.1	0.298	1.96	3.7	15
Nitrates	mg/kg d.w.	12	4980	111	2101	4912	8801	8	2612	88	423	2509	6606
FC	mg GAE/100 g f.w.	12	211	16	91	218	595	8	299	13	170	279	794
CUPRAC	µmol TE/100 g f.w.	12	2743	233	1320	2884	7988	8	4064	192	2464	3905	9779
Nitrites	µg/100 g f.w.	12	49	6.9	12	38	196	8	109	7.5	34	81	293
Nitrates	mg/100 g f.w.	12	90	1.97	45	90	153	8	54	1.997	11	49	134
DPPH	%	12	42	1.7	28	40	63	8	43	1.3	31	41	64

SD—standard deviation, Min—minimum, Max—maximum, n—number of products where analytes were determined >LOQ, d.w.—dry weight, f.w.—fresh weight, GAE—gallic acid equivalent, TE—Trolox equivalent.

**Table 3:** Results of TAC, TPC, nitrite, and nitrate content in the analysed DSs expressed per mass unit (g or kg) of product and daily dose (d. d.).

	Beetroot-Based Dietary Supplements																		
Method	Unit	Capsules						Tablets						Powders					
		n	Mean	SD	Min	Median	Max	n	Mean	SD	Min	Median	Max	n	Mean	SD	Min	Median	Max
FC	mg GAE/g	21	14	1.2	1.8	6.2	41	16	7.8	0.77	0.68	3.4	33	12	13	0.68	2.5	10	61
CUPRAC	µmol TE/g	21	126	11	13	62	312	16	76	3.5	1.3	42	278	12	139	5.9	27	119	467
Nitrites	µg/g	21	2.3	0.21	0.81	1.2	8.9	16	1.3	0.079	0.29	1.2	2.5	12	2.9	0.25	0.95	2.4	6.36
Nitrates	mg/kg	21	4230	155	383	2373	15,186	16	2099	67	504	1979	3746	12	4161	241	91	2265	13,110
FC	mg GAE/d. d.	21	15	1.3	0.73	8.5	42	16	17	1.4	1.5	18	41	12	99	9.6	4.1	92	251
CUPRAC	µmol TE/d. d.	21	138	10	5.2	103	363	16	167	8.1	5.5	174	350	12	1228	97	48	1065	3520
Nitrites	µg/d. d.	21	3.3	0.56	0.33	1.85	23.78	16	4.2	0.36	0.21	2.6	14	12	28	3.4	1.7	18	78
Nitrates	mg/d. d.	21	6.1	0.26	0.20	3.9	18	16	4.6	0.35	1.3	3.8	11	12	43	2.7	0.90	22	169
DPPH	%	21	39	1.67	7.2	22	90	16	35	2.4	4.9	17	90	12	37	1.3	12	37	90

SD—standard deviation, Min—minimum, Max—maximum, n—number of products where analytes were determined >LOQ, GAE—gallic acid equivalent, TE—Trolox equivalent, d. d.—daily dose recommended by the manufacturer.

**Table 4:** The realisation of ADI for NO_2_¯ and NO_3_¯ by DSs and beetroots.

		%ADI for NO_2_¯						%ADI for NO_3_¯				
Product	n	Mean	SD	Min	Median	Max	n	Mean	SD	Min	Median	Max
DSs and food products												
Tablets	14	0.030	0.030	0.0015	0.018	0.098	15	1.3	0.90	0.38	1.1	3.2
Capsules	18	0.023	0.039	0.0023	0.012	0.17	17	1.7	1.5	0.056	0.87	5.1
Powders	11	0.203	0.18	0.012	0.13	0.55	11	12	16	0.26	6.2	48
Beetroot												
Conventional	12	0.35	0.35	0.087	0.27	1.4	12	20.1	13	1.84	16.1	42.7
Organic	8	0.78	0.62	0.24	0.58	2.1	8	15	11	3.02	14	38

ADI for NO2¯ equals 0.2 mg of NO2¯/kg/day (14 mg of NO2¯/70 kg/day); ADI for NO3¯ equals 5 mg of NO3¯/kg/day (350 mg of NO2¯/70 kg/day); n—number of the analysed products with the determined content of analytes >LOQ.

**Table 5:** Summary of the labelling assessment of the analysed DSs.

The Analysed Feature of Product Marking	Results
Registration in the register of products is subject to notification of first placing on the market [39]	88% of analysed products were reported to the Register [39] and 12% did not
Labelling in Polish	26% of products had a complete or partial lack of markings in Polish
List of ingredients	5% of the packaging was missing the word “ingredients” before the list of ingredients
The net amount of food	26% of packages did not declare the net weight of the product
Date of minimum durability or best-before date	23% of manufacturers used incorrect wording preceding the date of minimum durability in the labelling
The presence of the term “dietary supplement”	6% of products were not marked with the term “dietary supplement”
Indication of the recommended daily portion	3% of products did not have the recommended daily portion for consumption specified
The presence of a warning regarding not exceeding the recommended daily portion	12% of packages lacked such a warning
The statement that DSs cannot be used as a substitute (replacement) for a varied diet	12% did not include this statement
The statement that DSs should be kept out of the reach of small children	9% did not include this statement
The content of vitamins and minerals and other substances with nutritional or other physiological effects present in the dietary supplement in numerical form, calculated per recommended daily portion of the product	21% of the packaging did not contain the content of vitamins, minerals and other substances per recommended daily portion
The information on the content of vitamins and minerals in percentage concerning the reference daily intake (RDI)	3% of the packaging did not contain information on the content of vitamins and minerals in percentage concerning RDI
Labelling may not suggest that the food has effects or properties that it does not have, or attribute to the food the property of preventing or treating disease	15% of the products suggested that the food had the properties of preventing or treating diseases
Health claims	21% of products contained health claims on the packaging that were not included in the EFSA Health Claims Register or were not allowed to be used due to a lack of scientific evidence

## Data Availability

Data is contained within the article or supplementary material.

## Supplementary Materials

The following supporting information can be downloaded at: https://www.mdpi.com/article/10.3390/foods12051017/s1, Table S1. Collected information about the analysed beetroot samples. Table S2. Collected information about the analysed beetroot-based DSs. Table S3. Optimisation of extraction of beetroot products on the example of a selected lyophilizate. Table S4. Optimisation of the FC method because of model and incubation time. Table S5. Optimisation of the FC method because of incubation time for lyophilizate and supplement in tablets according to method 1. Table S6. Full characteristics of the analysed beetroot-based dietary supplements because of TAC, nitrate, and nitrite content. Table S7. Full characteristics of the analysed beetroots samples because of TAC, nitrate, and nitrite content. Table S8. The results of the Mann-Whitney U test check the existence of differences between the individual groups of the analysed products (values *p* < 0.05 are marked in red). Table S9. The results of the correlation between the TAC and TPC results obtained by different methods of beetroot and beetroot–based products (Spearman’s rank correlation coefficient). Values which were statistically significant (*p* < 0.05) are marked in red. Table S10. Results of U Mann Whitney test for all analysed samples because of nitrites and nitrates content [mg/kg] (values *p* < 0.05 are marked in red). Table S11. The results of the correlation between the antioxidant potential and the content of nitrites and nitrate in beetroot and beetroot–based products (Spearman’s rank correlation coefficient). Values which were statistically significant (*p* < 0.05) are marked in red.
