# Supplementary material for: Antioxidant Capacity, Nitrite and Nitrate Content in Beetroot-Based Dietary Supplements
Source: Foods. 2023 Feb 27;12(5):1017. doi: 10.3390/foods12051017 (PMC10000616; doi:10.3390/foods12051017)
Supplement: Supplementary file 1 [file foods-12-01017-s001.zip › foods-2224291-supplementary.pdf]

Table S1. Collected information about the analysed beetroot samples.

| Form | Code | Water content (%) | Date of purchase | Certificate of organic cultivation | Place of purchase | Origin country |
|---|---|---|---|---|---|---|
| Conventionally cultivated | 1Bp | 85.9 | 11/05/2019 | no data | large-retail store, Gdańsk (PL) | Poland (PL) |
| | 1Bs | 83.2 | | | | |
| | 1Bu | 85.1 | | | | |
| | 3Bp | 85.2 | 11/14/2019 | no data | small-retail store, Kolbudy (PL) | PL |
| | 3Bs | 84.2 | | | | |
| | 3Bu | 84.2 | | | | |
| | 4Bp | 81.8 | 11/14/2019 | no data | large-retail store, Gdańsk (PL) | PL |
| | 4Bs | 78.4 | | | | |
| | 4Bu | 80.5 | | | | |
| | 5Bp | 88.1 | 11/28/2019 | no data | large-retail store, Gdańsk (PL) | PL |
| | 5Bs | 86.8 | | | | |
| | 5Bu | 87.8 | | | | |
| Organically cultivated | 2Bp | 83.2 | 11/05/2019 | P 095 18; Greater Poland (PL) | large-retail store, Gdańsk (PL) | PL |
| | 2Bu | 81.2 | | | | |
| | 6Bp | 85.2 | 12/02/2019 | PL-EKO-07-07904 Wilkowa Wieś (PL) | greengrocer's (online), Gdańsk (PL) | PL |
| | 6Bs | 80.9 | | | | |
| | 6Bu | 83.1 | | | | |
| | 7Bp | 83.4 | 12/02/2019 | PL-EKO-07-07904 Wilkowa Wieś (PL) | greengrocer's (online), Gdańsk (PL) | PL |
| | 7Bs | 80.0 | | | | |
| | 7Bu | 82.9 | | | | |

Bp – peeled beetroot; Bs – beetroot skins, Bu – unpeeled beetroot

Table S2. Collected information about the analysed beetroot-based DSs.

| Code | The content of beetroot extract or preserves/dosage unit | Declared weight of the dosage unit (g)[1] | Recommended daily dose | Origin country |
|---|---|---|---|---|
| C1 | beetroot extract (400 mg) containing 40 mg of nitrates; E464; inulin (70 mg) | NI / 0.3931 | 1x1 capsule | Poland (PL) |
| C2 | red beetroot extract (200 mg); young barley extract (200 mg); capsule (glazing agent: E464); vitamin $B_3$ (60 mg); vitamin $B_5$ (18 mg); E460; vitamin $B_2$ (4.2 mg); vitamin $B_6$ (4.2 mg ); vitamin $B_1$ (3.3 mg); vitamin $B_9$ (600 µg); vitamin $B_{12}$ (7.5 µg) | NI / 0.5151 | 1x1 capsule | PL |
| C3 | beetroot extract (550 mg); E1204 | NI / 0.5781 | 2x1 capsule | PL |
| C4 | extract of beetroot 10:1 (300 mg); capsule of vegetable cellulose | 0.39 / 0.4011 | 2x1 capsule | PL |
| C5A | beetroot concentrate (268 mg); ascorbic acid (20 mg); iron gluconate (12 mg); E1400; E470b; E551 | 0.376 / 0.3121 | 1x3 capsule | PL |
| C5B | | 0.376 / 0.3031 | | |
| C5C | | 0.376 / 0.301 | | |
| C6 | powdered red beetroot (400 mg); ascorbic acid (40 mg); iron (10 mg) | NI / 0.9961 | 1x2 capsule | PL |
| C7A | Beta vulgaris extract 4:1 (550 mg); pullulan capsule | 0.69 / 0.601 | 1x2 capsule | PL |
| C7B | | 0.69 / 0.6141 | | |
| C8A | dried juice concentrate (438.5 mg); ascorbic acid (38 mg); iron (2.8 mg); gelatine capsule | 0.596 / 0.5131 | 2x1 capsule with a meal | PL |
| C8B | | | | |
| C9 | freeze-dried juice from organic pickled beetroot (300 mg); micronized apple fibre; cellulose capsule | 0.3 / 0.2861 | 2x1 capsule before a meal | PL |
| C10 | 400 mg of root extract; 40 mg of nitrates | 0.5 / 0.3971 | 1x1 capsule | PL |
| C11 | 400 mg of root extract; 40 mg of nitrates | 0.5/ 0.4031 | 1x1 capsule | PL |
| C12 | beetroot (500 mg); E572; gelatine capsule | NI / 1.0141 | 3x2 caps | United States of America (USA) |
| C13 | organic beetroot extract with E1400 (700 mg) corresponding to 4620 mg of dried beetroot; HPMC capsule | NI / 1.4281 | 1x2 capsule with a meal | United Kingdom (UK) |
| C14 | beetroot (500 mg); E460; E551; E572; HPMC capsule | NI / 1.0781 | 3x2 capsule with a meal | USA |
| C15 | beetroot extract (500 mg); E460; E551; vegetable fatty; vegetable mineral salts | NI / 1.6251 | 3x1 capsule witch a meal | USA |

| Code | The content of beetroot extract or preserves/dosage unit | Declared weight of the dosage unit (g)[1] | Recommended daily dose | Origin country |
|---|---|---|---|---|
| C16 | beetroot extract (450 mg); E460; E464 capsule | NI / 1.1681 | 2x1 capsule | USA |
| C17 | beetroot extract 5:1 (500 mg) (5:1); E1400, E460; vegetable capsule shell: E464; anti-caking agents: E551, vegetable E572 | NI / 0.6281 | 1x1 capsule | USA |
| T1A | beetroot extract 20:1 (350 mg); E341; E470b | 0.35 | 1x2 tablet witch a meal | UK |
| T1B | | | | |
| T2A | beetroot concentrate (488 mg); ascorbic acid (20 mg); iron gluconate (12 mg); E1400; E470b; E551 | 0.65 | 1x3 tablet | PL |
| T2B | | | | |
| T2C | | | | |
| T3A | dried juice (500 mg) equals 3.5 g of raw vegetable; E470b; E551 | 0.925 | 1-2x3 tablet witch a meal | PL |
| T3B | | | | |
| T4 | dried juice concentrate (500 mg) equals 2.75 g of raw vegetable; $B_6$ (1 mg); $B_{12}$ (1.25 µg); E460; E470b, E551 | 0.55 | 1-2x3 tablet | PL |
| T5 | beetroot root powder (100 mg); L-arginine alpha-ketoglutarate (125 mg); L-citrulline (125 mg); Beta-alanine (100 mg); E421; E967; E960; E460; E466; HPMC capsule; E330; natural flavours (cherry and vanilla); E464; E470a; E551 | 1.42 | 1x1-2 tablet 20-30 min before a workout | USA |
| T6 | beetroot extract (80 mg); E341; E460; vegetable E572 (2.25 mg) | 0.35 | 2 tablet | UK |
| T7 | fresh beetroot 7:1 (500 mg); E1400; L-ascorbic acid (40 mg); 7 mg iron (II) fumarate (7 mg); E420; E470b; E551 | 0.63 | 1-2x1 tablet | PL |
| T8 | beetroot concentrate (500 mg); ascorbic acid (20 mg); iron (II) gluconate (12 mg); E1400; E470b; E551 | 0.65 | 3x1 tablet | PL |
| T9 | dried red beetroot concentrate (500 mg); vitamin $B_6$ (1 mg); vitamin $B_{12}$ (1.25 mg); E470b; E551 | 0.5 | 3x1-2 tablet | PL |
| T10 | beetroot extract (1000 mg; trimethylglycine); E460, vegetable fatty acids, E466, E470b; E464; E460ii | 1.46 | 1-3x1 tablet | USA |
| T11 | of beetroot extract 10:1 (160 mg) including 1600 mg of nitrates; E341; E460; E470b | 0.35 | 1x1-2 tablet with a meal | UK |
| T12 | beetroot extract 5:1 (315 mg); E341, E460; E570; E551; E572; E464; E422 ; E463 | NI / 0.821 | 1 tablet | UK |
| P1 | beetroot powder | 9.5 | 1x1 spoon | USA |
| P2 | 100% beetroot tuber extract | 4.0 | 3x1 teaspoon | Czech Republic (CZ) |
| P3 | powdered red beetroot | 3.0 | 1x1 teaspoon | Egypt (EG) |

| Code | The content of beetroot extract or preserves/dosage unit | Declared weight of the dosage unit (g)[1] | Recommended daily dose | Origin country |
|---|---|---|---|---|
| P4 | 100% powdered red beetroot | 15 | 1 teaspoon | Croatia (HR) |
| P5 | powdered BIO red beetroot | 15.0 | 1x3 teaspoons | Germany (DE) |
| P6 | powdered beetroot 4:1 (Beta vulgaris extract) | 1.0 | 1x half of a teaspoon | PL |
| P7 | powdered organic beetroot | 3.0 | 2-3 flat teaspoons | China (CN) |
| P8 | powdered red beetroot | 10 | 1 spoon | USA |
| P9 | beetroot crystals made of concentrated beetroot juice, 4 g/100 g of nitrates | 5.5 | 2-3 teaspoons, 1-3 hours before a workout or just after a workout | Ireland (IE) |
| P10 | 100% powdered red beetroot, whole ground, not peeled | 15 | Mix 1 tablespoon of the product with food | PL |
| P11 | powdered beetroot 600 mg; L-ascorbic acid (80 mg); iron fumarate (42 mg) | 0.72 | 1 measure (0.72 g) | NI |
| P12 | 100% powdered BIO red beetroot | 3.0 | Mix 1 teaspoon (3 g) of the product with 200 ml of food. The suggested daily dose is 1-2 teaspoons. | Italy (IT) |
| P13 | 100% powdered red beetroot | 10.0 | Add 1-2 teaspoons of beetroot powder (5-10 g) to food. | PL |

NI – lack of information; [1]for products where the manufacturer did not specify the weight of the unit and all products in capsules, it was determined empirically according to USP 43-NF 38 guidelines; <2091> chapter "Weight variation of dietary supplements". This value was used for calculations. Products with a lack of "dietary supplement" marking on the package were marked in orange in the Table.

Table S3. Optimisation of extraction of beetroot products on the example of a selected lyophilizate.

| | **Extraction technique** | **MAE** | | **UAE 1 (20ºC )** | | **UAE 2 (20ºC)** | |
|---|---|---|---|---|---|---|---|
| Variant | Solvent (v/v) | $\bar{x}$ [mg GAE/g] | RSD [%] | $\bar{x}$ [mg GAE/g] | RSD [%] | $\bar{x}$ [mg GAE/g] | RSD [%] |
| A | MiliQ+0.1%FA | 6.51 | 3.55 | 1.92 | 4.43 | 6.00 | 4.63 |
| B | 25%MeOH+0.1%FA | 7.23 | 3.20 | 2.39 | 6.75 | 7.15 | 5.78 |
| C | 50%MeOH+0.1%FA | 7.05 | 3.77 | 3.33 | 14.05 | 7.26 | 6.85 |
| D | 75%MeOH+0.1%FA | 5.75 | 3.89 | 6.37 | 5.00 | 5.82 | 1.38 |
| E | 25%EtOH+0.1%FA | 6.96 | 5.21 | 7.00 | 5.06 | 6.72 | 6.18 |
| F | 50%EtOH+0.1%FA | 6.65 | 5.10 | 7.04 | 7.06 | 6.95 | 0.021 |
| G | 75%EtOH+0.1%FA | 5.69 | 5.02 | 5.02 | 5.03 | 5.99 | 4.98 |

Table S4. Optimisation of the FC method because of model and incubation time.

| **Model** | **1** | | **2** | | **3** | | **4** | |
|---|---|---|---|---|---|---|---|---|
| Time of incubation [min] | $\bar{x}$ [Abs] | RSD [%] | $\bar{x}$ [Abs] | RSD [%] | $\bar{x}$ [Abs] | RSD [%] | $\bar{x}$ [Abs] | RSD [%] |
| 30 | 0.071 | 0.001 | 0.065 | 0.297 | 0.060 | 0.001 | 0.053 | 0.44 |
| 60 | 0.064 | 0.299 | 0.073 | 2.6 | 0.060 | 0.96 | 0.054 | 0.62 |
| 120 | 0.088 | 7.6 | 0.14 | 13 | 0.064 | 0.001 | 0.053 | 0.36 |

Table S5. Optimisation of the FC method because of incubation time for lyophilizate and supplement in tablets according to method 1.

| | **Time of incubation [min]** | $\bar{x}$ **[Abs]** | **RSD [%]** |
|---|---|---|---|
| lyophilizate | 30 | 0.324 | 2.7 |
| | 60 | 0.315 | 5.1 |
| | 120 | 0.312 | 4.2 |
| supplement | 30 | 0.247 | 5.6 |
| | 60 | 0.258 | 2.1 |
| | 120 | 0.262 | 13.6 |

Table S6. Full characteristics of the analysed beetroot-based dietary supplements because of TAC, nitrate, and nitrite content.

| Product | Daily dose [g] | FC | | CUPRAC | | DPPH | | Nitrites | | | Nitrates | | |
|---|---|---|---|---|---|---|---|---|---|---|---|---|---|
| | | mg GAE/g | SD | µmol TE/g | SD | Activity | SD | µg/g | SD | ADI[1] | mg/kg | SD | ADI[2] |
| T1A | 0.7 | 2.27 | 0.12 | 34 | 2.8 | 14% | 1.2 | 1.142 | 0.079 | 0.0057 | 3008 | 47 | 0.602 |
| T1B | 0.7 | 2.31 | 0.14 | 21 | 1.8 | 9.6% | 0.83 | 0.293 | 0.026 | 0.0015 | 3008 | 68 | 0.602 |
| T2A | 2.0 | 19.2 | 2.7 | 170 | 3.5 | 80% | 5.7 | 1.34 | 0.14 | 0.019 | 2569 | 65 | 1.4 |
| T2B | 2.0 | 16.25 | 0.34 | 175 | 7.6 | 88% | 1.5 | 2.00 | 0.29 | 0.028 | 1933 | 158 | 1.1 |
| T2C | 2.0 | 14.65 | 0.48 | 134 | 10 | 81% | 4.9 | 1.40 | 0.12 | 0.0195 | 2483 | 126 | 1.4 |
| T3A | 5.6 | 4.29 | 0.18 | 49 | 3.0 | 18% | 0.79 | 2.484 | 0.082 | 0.098 | 1624 | 117 | 2.6 |
| T3B | 5.6 | 4.10 | 0.46 | 49 | 1.2 | 16% | 1.0 | 2.24 | 0.26 | 0.089 | 1702 | 55 | 2.7 |
| T4 | 3.3 | 4.22 | 0.14 | 47 | 1.9 | 18% | 0.19 | 2.251 | 0.073 | 0.053 | 1979 | 159 | 1.9 |
| T5 | 2.8 | 1.30 | 0.15 | 38 | 5.5 | 8.9% | 0.98 | 0.877 | 0.079 | 0.018 | 504 | 60 | 0.41 |
| T6 | 0.7 | 2.11 | 0.64 | 21 | 0.15 | 18% | 0.34 | <LOQ | - | - | 3112 | 134 | 0.62 |
| T7 | 1.3 | 32.5 | 1.8 | 278 | 0.41 | 90% | 1.4 | 1.11 | 0.14 | 0.010 | 1050 | 75 | 0.38 |
| T8 | 2.0 | 13.0 | 1.5 | 134 | 12 | 81% | 8.8 | <LOQ | - | - | 2481 | 316 | 1.4 |
| T9 | 9.8 | 2.72 | 0.13 | 20 | 2.2 | 8.9% | 0.61 | 0.549 | 0.060 | 0.039 | 1142 | 140 | 3.2 |
| T10 | 4.4 | 0.681 | 0.085 | 1 | 0.52 | 4.9% | 0.57 | 0.537 | 0.082 | 0.017 | <LOQ | - | - |
| T11 | 0.7 | 2.415 | 0.056 | 34 | 4.1 | 11% | 0.89 | 1.20 | 0.16 | 0.0060 | 3746 | 91 | 0.75 |
| T12 | 2.5 | 2.40 | 0.20 | 18 | 2.4 | 13% | 1.1 | 0.721 | 0.023 | 0.013 | 1142 | 113 | 0.80 |
| C1 | 0.39 | 6.2 | 0.71 | 62 | 5.2 | 22% | 1.4 | 1.9 | 0.18 | 0.0053 | 4305 | 49 | 0.48 |
| C2 | 0.52 | 2.7 | 0.064 | 19 | 6.2 | 7.2% | 0.896 | 0.95 | 0.095 | 0.0035 | 383 | 13 | 0.056 |
| C3 | 1.2 | 3.7 | 0.22 | 46 | 3.9 | 15% | 0.52 | 3.2 | 0.25 | 0.026 | 1037.0 | 9.8 | 0.34 |
| C4 | 0.80 | 12 | 1.5 | 142 | 3.6 | 46% | 1.2 | 8.9 | 0.54 | 0.051 | <LOQ | - | - |
| C5A | 0.94 | 34 | 0.99 | 274 | 37 | 71% | 2.5 | 2.0 | 0.27 | 0.043 | 1959 | 49 | 3.5 |
| C5B | 0.91 | 35 | 2.0 | 254 | 4.8 | 90% | 0.97 | 0.88 | 0.084 | 0.0057 | 1926 | 36 | 0.50 |
| C5C | 0.90 | 31 | 2.8 | 278 | 13 | 90% | 1.6 | 1.2 | 0.13 | 0.0076 | 1907 | 128 | 0.49 |
| C6 | 1.0 | 37 | 3.4 | 285 | 27 | 82% | 7.2 | 2.8 | 0.26 | 0.020 | <LOQ | - | - |
| C7A | 1.2 | 3.6 | 0.25 | 44 | 1.1 | 14% | 0.38 | <LOQ | - | - | 5205 | 183 | 1.8 |
| C7B | 1.2 | 3.4 | 0.42 | 31 | 4.2 | 14% | 0.91 | 2.0 | 0.094 | 0.018 | 11924 | 576 | 4.2 |
| C8A | 1.0 | 41 | 3.2 | 268 | 26 | 74% | 1.5 | 1.8 | 0.078 | 0.013 | 3814 | 203 | 1.1 |
| C8B | 1.0 | 40 | 3.3 | 312 | 28 | 90% | 0.087 | 1.0 | 0.00302 | 0.0076 | 784 | 112 | 0.23 |
| C9 | 0.57 | 14 | 0.42 | 210 | 7.3 | 43% | 5.1 | 3.8 | 0.55 | 0.015 | 4739 | 125 | 0.77 |
| C10 | 0.40 | 1.96 | 0.10 | 13 | 1.8 | 11% | 1.5 | 0.93 | 0.0053 | 0.0026 | <LOQ | - | - |

| Product | Daily dose [g] | mg GAE/g | SD | µmol TE/g | SD | Activity | SD | µg/g | SD | ADI[1] | mg/kg | SD | ADI[2] |
|---|---|---|---|---|---|---|---|---|---|---|---|---|---|
| C11 | 0.40 | 1.8 | 0.039 | 16 | 1.1 | 10% | 0.49 | 0.81 | 0.1002 | 0.0023 | <LOQ | - | - |
| C12 | 3.0 | 3.2 | 0.16 | 33 | 3.0 | 12% | 1.0 | 0.99 | 0.082 | 0.022 | 1284 | 132 | 1.1 |
| C13 | 1.4 | 5.1 | 0.21 | 49 | 2.4 | 18% | 1.2 | 1.1 | 0.15 | 0.012 | 8461 | 256 | 3.5 |
| C14 | 3.2 | 8.7 | 1.02 | 112 | 1.9 | 33% | 0.82 | 7.4 | 0.81 | 0.170 | 4460 | 256 | 4.1 |
| C15 | 1.9 | 4.5 | 0.28 | 55 | 4.8 | 17% | 0.52 | 1.1 | 0.079 | 0.015 | 2373 | 218 | 1.3 |
| C16 | 1.2 | 8.9 | 0.11 | 100 | 6.4 | 41% | 0.50 | 1.2 | 0.081 | 0.010 | 15186 | 454 | 5.1 |
| C17 | 1.9 | 4.3 | 0.11 | 52 | 2.2 | 17% | 0.59 | <LOQ | - | - | 2170 | 50 | 1.2 |
| P1 | 9.5 | 9.9 | 0.21 | 127 | 9.8 | 37% | 1.5 | 2.2 | 0.18 | 0.15 | 1590 | 125 | 4.3 |
| P2 | 12 | 4.99 | 0.089 | 76 | 4.02 | 22% | 1.4 | 3.3 | 0.14 | 0.28 | 1774 | 1.2 | 6.1 |
| P3 | 3 | 10 | 0.19 | 105 | 12 | 40% | 1.6 | 2.5 | 0.37 | 0.053 | 299 | 41 | 0.26 |
| P4 | 15 | 11 | 0.55 | 119 | 2.5 | 34% | 2.2 | 0.95 | 0.078 | 0.10 | 1603 | 170 | 6.9 |
| P5 | 15 | 13 | 1.03 | 163 | 17 | 44% | 3.5 | 3.9 | 0.0088 | 0.42 | 1457 | 125 | 6.2 |
| P6 | 1 | 4.1 | 0.204 | 48 | 3.8 | 16% | 0.91 | 1.7 | 0.25 | 0.012 | 2757 | 316 | 0.79 |
| P7 | 9 | 6.8 | 0.66 | 80 | 1.6 | 24% | 1.2 | <LOQ | - | - | <LOQ | - | - |
| P8 | 10 | 9.2 | 0.48 | 106 | 4.99 | 31% | 0.22 | 1.2 | 0.17 | 0.089 | 91.4 | 1.6 | 0.26 |
| P9 | 17 | 15 | 2.1 | 213 | 15 | 48% | 1.6 | 1.7 | 0.12 | 0.21 | 10224 | 200 | 48 |
| P10 | 15 | 11 | 0.89 | 145 | 16 | 40% | 3.3 | 5.2 | 0.78 | 0.55 | 6597 | 495 | 28 |
| P11 | 0.72 | 61 | 2.0 | 467 | 12 | 90% | 1.1 | 2.8 | 0.034 | 0.014 | 5127 | 505 | 1.1 |
| P12 | 6 | 2.5 | 0.07995 | 27 | 1.2 | 12% | 0.29 | 2.3 | 0.29 | 0.099 | 5300 | 478 | 9.1 |
| P13 | 10 | 11 | 1.3 | 127 | 14 | 42% | 4.6 | 6.4 | 0.71 | 0.45 | 13110 | 755 | 37 |

[1] ADI calculated for 70 kg person (14 mg of nitrite ions/70 kg/day); [2]ADI calculated for 70 kg person (350 mg of nitrate ions/70 kg/day) SD—standard

Table S7. Full characteristics of the analysed beetroots samples because of TAC, nitrate, and nitrite content.

| | | | **FC** | | | | **CUPRAC** | | | | **DPPH** | | **Nitrites** | | | | | **Nitrates** | | | | |
|---|---|---|---|---|---|---|---|---|---|---|---|---|---|---|---|---|---|---|---|---|---|---|
| | Product | Daily dose [g f.w.] | mg GAE/g d.w. | SD | mg GAE/dose | SD | µmol TE/g d.w. | SD | µmol TE/dose | SD | Activity | SD | µg/g d.w. | SD | µg/dose | SD | ADI[1] | mg/kg d.w. | SD | mg/dose | SD | ADI[2] |
| Conventional | 1C | 100 | 11 | 0.95 | 187 | 17 | 146 | 3.9 | 2561 | 68 | 39% | 3.5 | 2.0 | 0.011 | 34 | 0.19 | 0.25 | 5645 | 388 | 99 | 6.8 | 28 |
| | 1Sk | 100 | 22 | 2.4 | 450 | 49 | 199 | 6.0 | 4012 | 122 | 63% | 6.2 | 3.2 | 0.26 | 66 | 5.3 | 0.47 | 7562 | 319 | 153 | 6.4 | 44 |
| | 1O | 100 | 7.2 | 0.15 | 117 | 2.5 | 93 | 2.7 | 1522 | 44 | 30% | 2.9 | 1.6 | 0.19 | 26 | 3.1 | 0.19 | 7307 | 129 | 120 | 2.1 | 34 |
| | 3C | 100 | 14 | 0.27 | 259 | 5.0 | 168 | 14 | 3144 | 256 | 41% | 0.27 | 1.4 | 0.0017 | 26 | 0.032 | 0.19 | 2908 | 230 | 55 | 4.3 | 16 |
| | 3Sk | 100 | 26 | 1.7 | 482 | 33 | 365 | 43 | 6876 | 815 | 60% | 1.3 | 2.5 | 0.0026 | 47 | 0.049 | 0.33 | 2535 | 115 | 48 | 2.2 | 14 |
| | 3O | 100 | 12 | 0.46 | 202 | 8.1 | 151 | 9.1 | 2624 | 159 | 42% | 0.27 | 0.702 | 0.055 | 12 | 0.96 | 0.087 | 2570 | 142 | 45 | 2.5 | 13 |
| | 4C | 100 | 12 | 0.23 | 291 | 5.5 | 150 | 3.7 | 3623 | 89 | 40% | 2.1 | 1.7 | 0.14 | 42 | 3.4 | 0.30 | 4178 | 243 | 101 | 5.9 | 29 |
| | 4Sk | 100 | 22 | 1.8 | 595 | 51 | 289 | 25 | 7988 | 684 | 56% | 2.7 | 7.1 | 0.91 | 196 | 25 | 1.4 | 2101 | 166 | 58 | 4.6 | 17 |
| | 4O | 100 | 8.0 | 1.3 | 179 | 28 | 110 | 11 | 2445 | 241 | 32% | 3.1 | 2.0 | 0.12 | 45 | 2.7 | 0.32 | 6692 | 141 | 149 | 3.1 | 43 |
| | 5C | 100 | 11 | 1.3 | 147 | 17 | 145 | 9.4 | 2008 | 131 | 37% | 3.0 | 1.6 | 0.23 | 22 | 3.2 | 0.16 | 8801 | 287 | 122 | 4.0 | 35 |
| | 5Sk | 100 | 15 | 0.94 | 235 | 14 | 260 | 18 | 3945 | 274 | 52% | 3.2 | 3.4 | 0.023 | 51 | 0.35 | 0.37 | 3456 | 13 | 52 | 0.19 | 15 |
| | 5O | 100 | 6.8 | 0.32 | 91 | 4.3 | 98 | 3.7 | 1320 | 50 | 28% | 2.3 | 1.4 | 0.17 | 19 | 2.3 | 0.13 | 6003 | 352 | 82 | 5.9 | 23 |
| Organic | 2C | 100 | 12 | 0.49 | 284 | 11 | 160 | 5.4 | 3712 | 126 | 39% | 1.1 | 3.8 | 0.55 | 89 | 13 | 0.64 | 3511 | 255 | 134 | 3.3 | 23 |
| | 2O | 100 | 9.7 | 0.64 | 196 | 13 | 123 | 13 | 2490 | 265 | 36% | 2.5 | 3.6 | 0.53 | 73 | 11 | 0.52 | 6606 | 162 | 57 | 0.38 | 38 |
| | 6C | 100 | 13 | 1.1 | 275 | 23 | 208 | 11 | 4244 | 216 | 46% | 0.57 | 2.0 | 0.14 | 41 | 2.9 | 0.30 | 2817 | 19 | 11 | 0.28 | 16 |
| | 6Sk | 100 | 34 | 0.91 | 794 | 22 | 413 | 15 | 9779 | 366 | 63% | 3.3 | 2.8 | 0.087 | 66 | 2.1 | 0.47 | 461 | 12 | 39 | 0.48 | 3.1 |
| | 6O | 100 | 9.8 | 0.68 | 170 | 12 | 151 | 6.9 | 2613 | 121 | 37% | 0.82 | 2.0 | 0.29 | 34 | 5.0 | 0.24 | 2235 | 28 | 58 | 2.4 | 11 |
| | 7C | 100 | 15 | 0.93 | 310 | 19 | 198 | 4.3 | 4097 | 89 | 44% | 1.3 | 4.8 | 0.44 | 100 | 9.1 | 0.72 | 2782 | 116 | 11 | 0.30 | 16 |
| | 7Sk | 100 | 24 | 1.8 | 600 | 45 | 358 | 28 | 8933 | 698 | 64% | 5.5 | 7.0 | 1.0 | 175 | 26 | 1.3 | 423 | 12 | 41 | 1.5 | 3.02 |
| | 7O | 100 | 9.9 | 0.72 | 198 | 14 | 124 | 16 | 2464 | 309 | 31% | 2.6 | 15 | 0.59 | 293 | 12 | 2.1 | 2065 | 75 | 82 | 5.9 | 12 |

[1] ADI calculated for 70 kg person (14 mg of nitrite ions/70 kg/day); [2]ADI calculated for 70 kg person (350 mg of nitrate ions/70 kg/day); d.w. – dry weight

Table S8. The results of the Mann-Whitney U test check the existence of differences between the individual groups of the analysed products (values $p < 0.05$ are marked in red).

| | | | **Tablets** | **Capsules** | **Powders** | **Lyophilizate** |
|---|---|---|---|---|---|---|
| **F-C assay** | Expressed as mg GAE/d.d. | Tablets | - | 0.987924 | $9.9\times10^{-5}$ | $3\times10^{-10}$ |
| | | Capsules | 0.987924 | - | $4.4\times10^{-5}$ | $5\times10^{-8}$ |
| | | Powders | $9.9\times10^{-5}$ | $4.4\times10^{-5}$ | - | $3.8\times10^{-5}$ |
| | | Lyophilizate | $3\times10^{-10}$ | $5\times10^{-8}$ | $3.8\times10^{-5}$ | - |
| | Expressed as mg GAE/g | Tablets | - | 0.072627 | 0.07546 | $4.866\times10^{-3}$ |
| | | Capsules | 0.072627 | - | 0.483821 | 0.146878 |
| | | Powders | 0.07546 | 0.483821 | - | 0.093978 |
| | | Lyophilizate | 0.004866 | 0.146878 | 0.093978 | - |
| **CUPRAC** | Expressed as mg TE/ d.d. | Tablets | - | 0.617087 | $1.59\times10^{-4}$ | $3\times10^{-10}$ |
| | | Capsules | 0.617087 | - | $2.3\times10^{-5}$ | $5\times10^{-8}$ |
| | | Powders | $1.59\times10^{-4}$ | $2.3\times10^{-5}$ | - | $7\times10^{-6}$ |
| | | Lyophilizate | $3\times10^{-10}$ | $5\times10^{-8}$ | $7\times10^{-5}$ | - |
| | Expressed as mg TE/g | Tablets | - | 0.138403 | 0.055535 | $2.53\times10^{-4}$ |
| | | Capsules | 0.138403 | - | 0.440962 | 0.034565 |
| | | Powders | 0.055535 | 0.440962 | - | 0.024274 |
| | | Lyophilizate | $2.53\times10^{-3}$ | 0.034565 | 0.024274 | - |

d. d. – daily dose recommended by the manufacturer

Table S9. The results of the correlation between the TAC and TPC results obtained by different methods of beetroot and beetroot–based products (Spearman's rank correlation coefficient). Values which were statistically significant ($p < 0.05$) are marked in red.

| | | **FC [mg GAE/g]** | **CUPRAC [µmol TE/g]** | **DPPH [%]** |
|---|---|---|---|---|
| Beetroot | **FC [mg GAE/g]** | x | 0.960787 [b] | 0.957410 [b] |
| | CUPRAC [µmol TE/g] | 0.960787 [b] | x | 0.940955 [b] |
| | DPPH [%] | 0.957410 [b] | 0.940955 [b] | x |
| Capsules | **FC [mg GAE/g]** | x | 0.971429 [b] | 0.968831 [b] |
| | CUPRAC [µmol TE/g] | 0.971429 [b] | x | 0.971429 [b] |
| | DPPH [%] | 0.968831 [b] | 0.971429 [b] | x |
| Tablets | **FC [mg GAE/g]** | x | 0.858824 [b] | 0.850000 [b] |
| | CUPRAC [µmol TE/g] | 0.858824 [b] | x | 0.852941 [b] |
| | DPPH [%] | 0.850000 [b] | 0.852941 [b] | x |
| Powders | **FC [mg GAE/g]** | x | 0.923077 [b] | 0.939560 [b] |
| | CUPRAC [µmol TE/g] | 0.923077 [b] | x | 0.917582 [b] |
| | DPPH [%] | 0.939560 [b] | 0.917582 [b] | x |

[a] $p > 0.05$; [b] $p < 0.05$

Table S10. Results of U Mann Whitney test for all analysed samples because of nitrites and nitrates content [mg/kg] (values $p < 0.05$ are marked in red).

| | | **Beetroot** | **Capsules** | **Tablets** | **Powders** |
|---|---|---|---|---|---|
| Nitrites | beetroot | x | 0.014038 | $2.95\times10^{-4}$ | 0.671316 |
| | capsules | 0.014038 | x | 0.262612 | 0.118561 |
| | tablets | $2.95\times10^{-4}$ | 0.262612 | x | $7.429\times10^{-3}$ |
| | powders | 0.671316 | 0.118561 | $7.429\times10^{-3}$ | x |
| Nitrates | beetroot | x | 0.262612 | 0.262612 | 0.262612 |
| | capsules | 0.262612 | x | 0.724077 | 0.722644 |
| | tablets | 0.262612 | 0.724077 | x | 0.59859 |
| | powders | 0.262612 | 0.722644 | 0.59859 | x |

Table S11. The results of the correlation between the antioxidant potential and the content of nitrites and nitrate in beetroot and beetroot–based products (Spearman's rank correlation coefficient). Values which were statistically significant ($p < 0.05$) are marked in red.

| | | **Nitrites [µg/g]** | **Nitrates [mg/kg]** |
|---|---|---|---|
| beetroot | FC [mg GAE/g] | 0.398041 [a] | -0.536753 [b] |
| | CUPRAC [µmol TE/g] | 0.409929 [a] | -0.628808 [b] |
| capsules | FC [mg GAE/g] | 0.423514 [a] | 0.085994 [a] |
| | CUPRAC [µmol TE/g] | 0.426762 [a] | $8.469 \times 10^{-3}$ [a] |
| tablets | FC [mg GAE/g] | 0.484180 [a] | 0.038235 [a] |
| | CUPRAC [µmol TE/g] | 0.482708 [a] | 0.067647 [a] |
| powders | FC [mg GAE/g] | 0.202797 [a] | 0.195804 [a] |
| | CUPRAC [µmol TE/g] | 0.272727 [a] | 0.209790 [a] |

[a] $p > 0.05$; [b] $p < 0.05$
